# Genomics-informed approach identifies which cell types regulate the metabolome

**DOI:** 10.1093/bioinformatics/btag330

**Published:** 2026-06-02

**Authors:** Haim Krupkin, Evin M Padhi, Daniel Nachun, Jessica Kain, Jonathan Z Long, Stephen B Montgomery

**Affiliations:** Department of Genetics, Stanford University School of Medicine, Stanford, CA 94305, United States; Department of Pathology, Stanford University School of Medicine, Stanford, CA 94305, United States; Department of Pathology, Stanford University School of Medicine, Stanford, CA 94305, United States; Department of Pathology, Stanford University School of Medicine, Stanford, CA 94305, United States; Department of Genetics, Stanford University School of Medicine, Stanford, CA 94305, United States; Department of Pathology, Stanford University School of Medicine, Stanford, CA 94305, United States; Sarafan ChEM-H, Stanford University, Stanford, CA 94305, United States; Department of Genetics, Stanford University School of Medicine, Stanford, CA 94305, United States; Department of Pathology, Stanford University School of Medicine, Stanford, CA 94305, United States

## Abstract

**Motivation:**

Metabolism occurs in a cell type-specific manner, but which cells regulate metabolite levels remains unclear.

**Results:**

Here, we integrate some of the largest metabolite quantitative trait loci datasets, TOPMed and UK Biobank, with one of the most extensive single-cell RNA sequencing resources, Tabula Sapiens. This integration allows us to identify cell types that regulate metabolites body-wide. We find hepatocytes are the primary regulatory cell type for most metabolites, associating with 385/410 (94%) metabolites for whom an association is found. Additionally, our multi-gene approach reveals more metabolite associations with beta cells compared to those identified using a single-gene approach. For example, we identify novel metabolite-cell type associations, such as the association between phenylpropanoic acid and beta cells, this metabolite that was previously thought to be regulated by the microbiome.

**Availability:**

Code used in this work is available via Github at https://github.com/haimkru/Metabolite-Cell-Type-Associations.

## 1 Main

**Figure 1:**
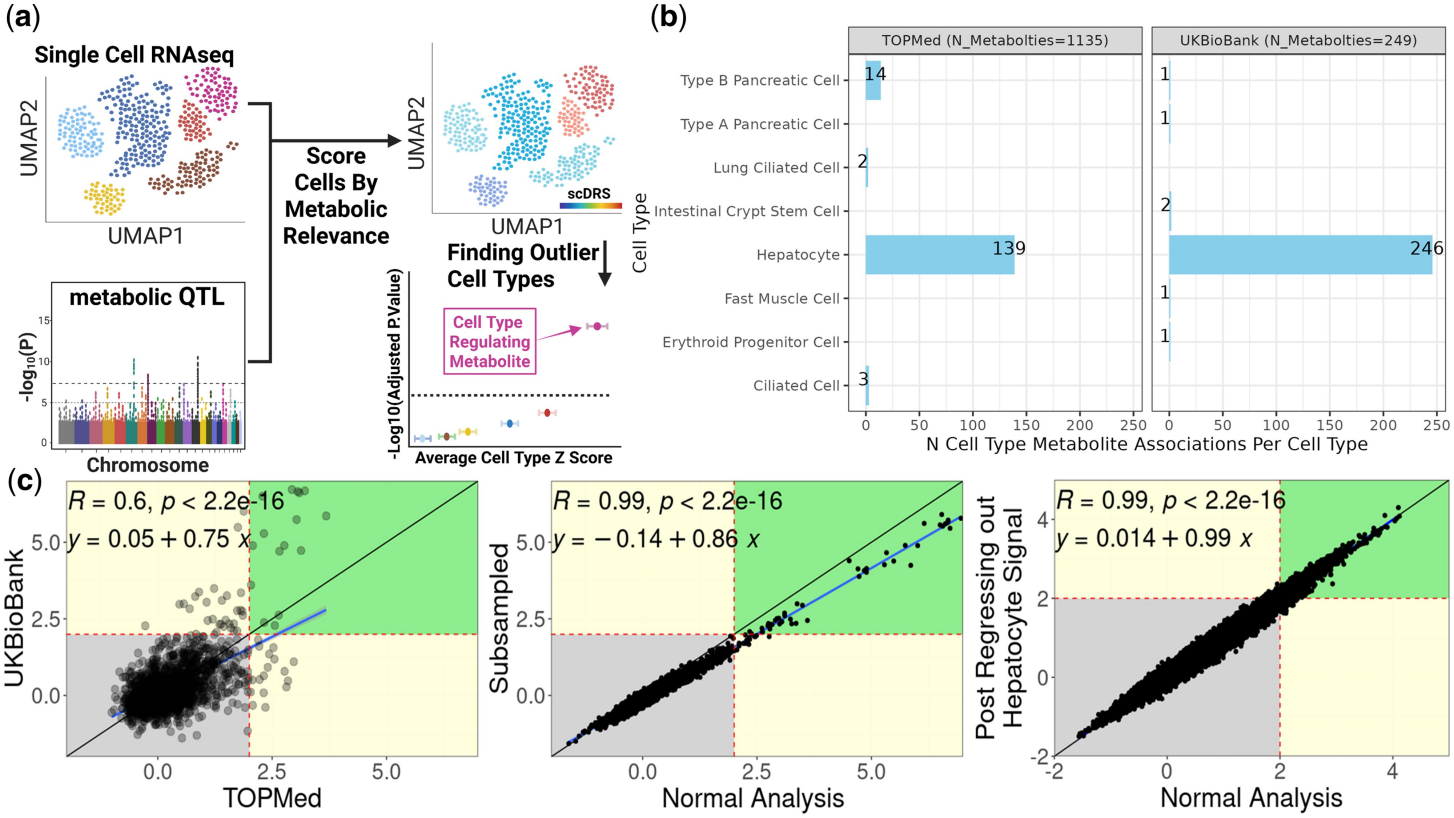
(a) Schematic of workflow. We start with metQTLs and whole- body scRNAseq data. We then convert metQTLs into metabolite-associated genes (metGenes) using MAGMA (De Leeuw *et al*. 2015), and use these metGenes to run scDRS (Zhang *et al*. 2022). Then, we identify cell types whose scDRS scores are consistently outliers. The rationale being cell types with outlier scores likely influence metabolite levels (Methods 2.1). (b) Bar plot representing the number of metabolites associated with a cell type in the analyses performed using TOPMed (left) and UK Biobank (right) metQTLs. (c) Some of the robustness checks for our workflow Both X axis and Y axis represent the average Z score of a metabolite-cell type association. (Left) Comparison of association results for metabolites analyzed in both data sources, derived from analyses conducted using TOPMed and UK Biobank. (Middle) Due to the high computational cost of running our workflow on the 500 000 cells in Tabula Sapiens, we also tried running our analyses on 70 cells from each cell type. This analysis was done using UK Biobank metQTL data. (Right) Comparison of the metabolite-cell type association results before and after regressing out the hepatocyte signal. This analysis was done using UK Biobank metQTL data integrated with Tabula Sapiens Adult scRNAseq.

Metabolites are small molecules that play essential roles in various biological processes as well as human diseases (Shi *et* *al.* 2023), and have long been known to be regulated by specific cell types (Gebert *et al.* 2021). However, for many metabolites, we do not know which cell types regulate them. One could identify which cell types regulate metabolites from conditions that emulate a permutation experiment. An example of these conditions is type 1 diabetes, in which the lack of pancreatic beta cells leads to dysregulation of glucose homeostasis. But these conditions are often metabolically complex, and involve multiple cell types and feedback loops. For such reasons, there is little known about which specific cell types regulate many metabolites. To close this gap in identifying cell types that regulate metabolites, we examined some of the largest trans metabolic quantitative trait loci (metQTL) studies TOPMed (Wang *et al.* 2025) and UK Biobank (Tambets *et al.* 2026), representing 15 485 and 619 372 participants accordingly, integrated with one of the largest body wide single-cell RNA sequencing (scRNA-seq) studies, Tabula Sapiens (The Tabula Sapiens Consortium 2022) (Fig. 1a). This method operates on the premise that an outlier enrichment of metabolite-associated genes within a specific cell type, when compared to all other cell types, reflects a cell type’s regulatory role (Methods 2.1). We used the UK Biobank metQTL data for analyses wherein maximizing statistical power was prioritized, and the TOPMed metQTL data whenever broader diversity of metabolites was desired.

Using our approach, we found hepatocytes were the cell type associated with the most metabolites (385/410 94% of all identified associations), beta cells were also a vital regulator of the metabolome (14/410, 3.4%) (Fig. 1b; Tables S1 and S2). To ensure the validity of this approach, we also establish its independence from metQTL source (Fig. 1c left; Fig. S1). We theorize that a larger metQTL cohort size increases the average genes’ statistical significance used in our multi-gene analysis, which might give rise to the increase in number of associated cell types (Fig. S1 and S2a). We also examined the effects of linkage disequilibrium reference ancestry, quality of the cells found in the scRNAseq, number of metGenes used (we denote hereafter metGene as genes associated with a metabolite) (Fig. S2b–d), and number of cells per cell type (Fig. 1c middle). Although the ranking of cell types’ association with the metabolite is unchanged by these variables, reducing the number of genes or cells decreases the signal-to-noise ratio, resulting in weaker association of significant findings. A limitation of the aforementioned robustness assessments is that the strong correlations observed across our associations permutation analyses might largely be driven by a few significant outlier associations (e.g. hepatocytes). Thus, our evaluation metrics are not well-suited to evaluate the robustness of association strengths among non-significant hits.

**Figure 2:**
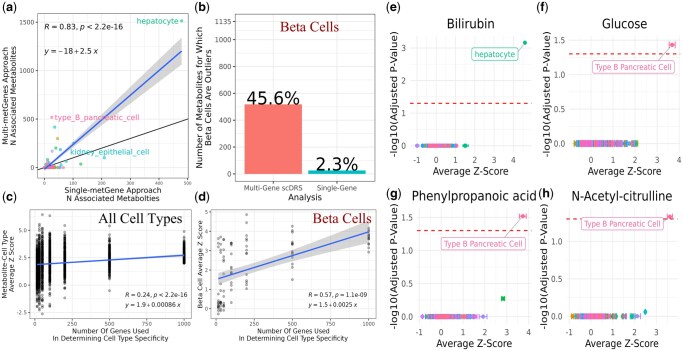
Comparison between the number of associated metabolites per cell type using a single-gene or a multi-gene approach. (a) Across all cell types, (b) in beta cells specifically. Examination of the affect of increasing the number of genes used in the multi-gene approach in measuring the cell type’s average Z score: (c) Across all cell types, (d) in beta cells specifically. Scatter plots visualizing association results between metabolites and cell types, each plot representing a metabolite, and each dot representing a cell type with whom the metabolite is tested for association. The y-axis represents the *P* value of the association after adjustment for multiple testing, and the x-axis represents the association’s average Z score in the cell type. Threshold represents adjusted *P* value <0.05. Known associations: (e) Glucose, (f) Bilirubin. Novel associations: (g) Phenylpropanic acid, (h) N-acetyl-citrulline. *This analysis was done using TOPMed metQTL data integrated with Tabula Sapiens Adult scRNAseq.

Given the high incidence of metabolite-hepatocyte associations, all signals were evaluated for potential obscuring by hepatocyte signals. Such obscurement could occur from technical factors or from signal masking, wherein lead signals can hide secondary signals (Hadi and Simonoff 1993). Therefore, we attempted to retrieve secondary signals by removing the hepatocyte signal in multiple ways (Methods 2.3). Specifically, we tried to regress out the hepatocyte signal (Fig. 1c right).

Also, as the hepatocyte signals might mask secondary outliers signals due to shifting the distribution to the right, we also just “removed” the hepatocyte cells from the analysis (Fig. S3a). Finally, to ensure the hepatocyte signal isn’t a result of a few hepatocyte marker genes, overwhelming our analysis, we also redid the analysis removing all hepatocyte marker genes, resulting in the same result (Fig. S3b). Both the default, as well as the methods to mask the hepatocyte signal, yielded similar metabolite-cell type associations (excluding for hepatocytes, which were, as expected, altered).

We also compared our metabolite-cell type association approach to a simpler single-gene method. Specifically, we evaluated how our method performed relative to directly identifying cell-type outliers based solely on individual metGenes obtained from the strongest metQTL signals (Methods 2.4). Similarly to the results from our multi-gene approach, hepatocytes were again the cell type associated with the most metabolites (Fig. 2a; Fig. S5a). Despite the well-established metabolic roles of beta cells, the single-gene approach identified fewer metabolite associations for beta cells and did not detect some known associations, such as the glucose-beta cells association (Banting *et al.* 1922) (Fig. 2b). This phenomenon may occur because beta cells are more easily identified as outliers when the analysis includes more genes. This is supported by the increased slope and variance explained as the number of genes increases in beta cells compared to all cells (Fig. 2c and d). Further, our multi-gene analysis identified both known and novel associations between metabolite levels and cell types. Specifically, we reconfirmed the previously established links between bilirubin and hepatocytes (Fig 2e), and glucose’s association with pancreatic beta cells (Fig 2f). Both of these known metabolite-cell type associations are widely recognized due to their central roles in physiology. For example, the failure of pancreatic beta cells to properly regulate blood glucose levels is the hallmark of diabetes, while the inability of damaged hepatocytes to process and clear bilirubin leads to its accumulation, manifesting as hepatic jaundice (Banting *et al.* 1922, Ashcroft and Rorsman 2012, Sticova and Jirsa 2013, Erlinger *et al.* 2014). In addition, we also discovered some novel associations, such as the associations of phenylpropionic acid (PPA) (Fig 2g) with pancreatic beta cells (Liberzon *et al.* 2015). In the human body, dietary L-phenylalanine is metabolized into PPA by a gut microbiome enzyme. PPA is then absorbed into the body and converted into phenylpropioglycine by hepatic glycine N-acyltransferase (Sticova and Jirsa 2013). PPA’s association with beta cells is of special interest because it, and it’s derivatives, have been reported to influence fatty-acid and glucose metabolism, as well as inflammatory signaling. Thus showing promise as potential therapeutics (Kinra *et al.* 2021, Cho *et al.* 2023, Alqudah *et al.* 2025). Further study into PPA’s association with beta cells could help elucidate its mechanisms of action. Using the multi-gene approach we also find a new association between beta cells and N-acetyl-citrulline (Fig 2h). N-acetyl-citrulline is a proposed biomarker for prostate cancer (Chen *et al.* 2025), and thus understanding its association with beta cells could be beneficial. Overall, these findings demonstrate the utility of this approach for revealing novel insights into metabolic regulation.

The single-gene approach complements the multi-gene approach. For instance, it successfully identified some known metabolite-cell type associations. Specifically, we reconstructed the tryptophan-kynurenine pathway in langerhans cells, identifying outlier expression of the rate-limiting enzyme IDO1 and the potential tryptophan transporter SLC16A10 (Mariotta *et al.* 2012, Krupa and Kowalska 2021) (see Fig. S5b and c). We also find some suspected novel associations, such as between N-acetyl-citrulline and kidney epithelial cells (Fig. S5d; Table S4). The observed differences in the associations found by the multi-gene versus the single-gene approach might reflect a difference in regulation scheme of the metabolites. The single-gene approach might be particularly well-suited for capturing metabolite-cell type associations when a metabolite’s metabolism steps are performed by different genes across different cell types. Complementarily, a multi-gene approach might be well suited when a metabolite is governed by multiple genes all operating within a singular cell type.

In addition to examining metabolite-cell type associations in adults, we also investigated metabolite-cell type associations in human fetal samples (Cao *et al.* 2020) (Methods 2.5). Although fetal associations were largely similar to those observed in adults (Fig. S6a–b and d), the strength of associations was generally reduced. The reduced association strengths in fetal cell types may stem from using adult metQTLs; a similarly powered study using fetal metQTLs might yield stronger associations. Additionally, maternal metabolic support might naturally lower metabolite regulation by the fetus (Lowe *et al.* 2017). Moreover, we identified an association between the placenta and lactic acid (Fig. S6c; Table S3). We highlight that this association would have been missed had placental tissues not been sampled. This emphasizes the importance of including placental cell types in future studies, especially when studying metabolism in a cell type-specific manner.

The current study has several limitations. First, our use of the Tabula Sapiens scRNAseq dataset excludes brain cell types, which are known to be important in metabolism (Camandola and Mattson 2017). Thus, we may miss metabolite associations specific to brain cells or incorrectly attribute such associations to expressionly similar peripheral cell types. Second, our approach relies on outlier analysis, which assumes the cell type regulating a metabolite represents a minority (<5%) of sampled cells. This assumption limits our ability to detect associations driven by more abundant cell types or multiple cell types collectively exceeding this threshold. Additionally, our method is biased by way of relaying on predefined cell type annotations and assumes intra-cell-type homogeneity, potentially missing associations driven by subpopulations or other cellular characteristics such as cell cycle stage. Third, our analysis relies on metQTLs to identify metGenes, inherently excluding highly conserved genes lacking detectable genetic variation. Furthermore, the TOPMed metQTL dataset, although comprehensive (1135 metabolites), is insufficiently powered. This is shown by the higher number of associations per metabolite identified in the larger UK Biobank metQTL cohort compared to the number of associations for the same metabolites identified from the smaller TOPMed metQTL cohort. Finally, our work suggests critical cell types for metabolism that remain to be explored with future biochemical assays.

In conclusion, our genomics-based systematic integration approach successfully recapitulates known associations, such as pancreatic beta cells regulating glucose and hepatocytes influencing bilirubin. Additionally, this method uncovers novel relationships between cell types and blood metabolites, highlighting previously unrecognized regulatory roles. Notably, we identified novel roles for beta cells in regulating specific metabolites. These findings were robust across different metQTL data sources, including TOPMed and UK Biobank targeted metQTL studies. Moreover, we emphasized the importance of sampling across developmental stages, as without doing so, some associations would be missed, such as the association between lactic acid and the placenta. Overall, our results provide a foundational resource for understanding cell type specific metabolic regulation. Highlighting new biological insights and potential avenues for future research into human metabolism and disease.

## 2 Methods

### 2.1 Multi-gene outlier analysis for metabolite cell types

The term “regulate” is used to describe a cell type’s ability to alter the levels of a metabolite in the blood through multiple mechanisms, including directly, such as through enzyme activity and transport, and indirectly, such as through hormonal control of its metabolism. To identify regulatory cell types of metabolites, we applied the scDRS tool (Zhang *et al.* 2022) using data derived from trans-metQTLs. Because each metabolite is modeled independently when testing for cell type associations, our approach avoids many of the limitations of multiple metabolites based pathway enrichment analyses (Lee *et al.* 2025). Our analysis was done using trans-metQTLs. An alternative method would be to use cis-eQTLs with genes known to be associated with the metabolite. trans-metQTL data was used instead, as trans-metQTLs are more unbiased and scale better as they does not require prior gene metabolic role annotations. Nonetheless, as all genome wide association studies, this trans-QTL approach’s application is limited for detecting highly conserved metGenes controlling metabolite levels, as selection keeps functional variation rare in these genes (Glassberg *et al.* 2019). Importantly, scDRS uses MAGMA to aggregate individual metQTL summary statistics to gene level summary statistics (De Leeuw *et al.* 2015). Following our application of scDRS we transformed the normalized scores from scDRS for each cell for each metabolite into z scores across the entire scRNA-seq dataset, then calculated both the average z score and the standard error for each cell type. We then converted these average Z scores into P. values, retrieving the cell type-metabolite association’s raw P. value. Similar metabolite-cell type association tests often yield comparable association strengths due to shared biological mechanisms, treating them as strictly independent artificially inflates the rate of false negatives. To mitigate this, we adjusted raw P. values using the eigenvalue-based method proposed by Li and Ji (2005). Specifically, we constructed a correlation matrix of the metabolite-cell type associations and extracted its eigenvalues to calculate the effective number of independent tests ($M_{\mathrm{eff}}$). This step quantifies the dataset’s true dimensionality by effectively collapsing redundant metabolite-cell type association signals. Ultimately, using $M_{\mathrm{eff}}$ instead of the raw test count or an overly conservative Bonferroni correction, allowed for a high level of conservativeness while taking into account the similarity between different metabolites and cell types. A metabolite-cell type association was considered significant, only if its adjusted P. value <0.05. However, we suspect that there may be many false-negative associations, wherein the average z score for the cell type-metabolite association exceeds 1.96, yet the adjusted P. value remains >0.05 due to the conservativeness of our approach. Importantly, this pipeline is non-directional at both steps: MAGMA’s gene test (F-test) captures overall variance explained, without preserving the sign of SNP effects (De Leeuw *et al.* 2015), and scDRS evaluates whether a cell preferentially expresses the associated gene set (Zhang *et al.* 2022). Thus, our results implicate cell types enriched for expression of metabolite-associated genes (metGenes), but do not infer the direction of effect on metabolite abundance. Moreover, it is limited in capturing metabolic regulation through non-transcriptional means, and can not estimate the variance in metabolite levels explained by the contribution of each cell type.

### 2.2 Statistics and reproducibility

All statistical tests were performed in R (version 4.2.2) and Python (version 3.10.9). Statistical tests include Wilcoxon test, Pearson correlation test, students’ *t* test. All tests were two-sided, and *P* values <0.05 were considered significant. Randomization and blinding were not feasible for this study because it utilized observational data.

### 2.3 Hepatocyte masking

Our outlier-based analysis indicated that hepatocytes might overshadow the influence of secondary regulatory cells. As due to their high average Z score, they could potentially mask secondary regulatory cell types (Hadi and Simonoff 1993). Moreover, a hepatocyte metabolite such as bilirubin might be cross contaminating other metabolites, thus also masking non-hepatocyte associations (Kroll and Elin 1994). To test these possibilities, we employed three different reanalysis strategies. First, we excluded hepatocytes from our analysis after all cells were scDRS scored. Second, when performing the scDRS scoring, after MAGMA (De Leeuw *et al.* 2015) scoring, we excluded all hepatocyte gene markers from the investigation, which we identified by using Seurat’s default FindMarkers command (Satija *et al.* 2015) as well as PangloDB’s hepatocyte cell markers (Franzén *et al.* 2019). Third, we applied a partial regression to remove the effect of hepatocytes’ signal from the other cell types’ associations. Wherein, we fit a distinct linear regression model for each individual cell type. In each model, the regression was calculated across all metabolites simultaneously, with the given metabolite-cell type’s average z-scores acting as the dependent variable (*y*) and the hepatocyte-metabolite average z-scores acting as the independent variable (*x*). From each model, we extracted the resulting regression slope (β), which quantifies the magnitude of the hepatocyte correlation. We then performed a partial residualization. Crucially, the raw slope was not subtracted from the data; rather, we calculated the corrected average z-scores by subtracting the hepatocyte average z-scores multiped by the extracted slope from correlation with the other cell type’s average z-scores. For example, if the regression determined a slope of β=0.5 between cell type C and hepatocytes, and the original average z-score for Metabolite M was 4.0 in hepatocytes and 3.0 in Cell Type C, the corrected score for cell type C is calculated by subtracting the scaled hepatocyte signal (4.0×0.5=2.0) from the original cell type C signal (3.0−2.0=1.0). This is analogous to the covariate removal methodology utilized in the *limma* package (Ritchie *et al.* 2015).

### 2.4 Identifying metabolic genes

To find genes that associate with the levels of a specific metabolite, such as by producing it or transporting it, we used data from metQTLs. Specifically, we focused on significant variants ($p.\text{value}<5\times 10^{-8}$), and aimed to assign each metQTL to a gene. Accurately assigning genetic variants to genes is a known challenge. To address this challenge, we explored multiple different approaches of converting metQTLs to metGenes, and chose the one with the best performance, as indicated by its metabolic gene ontologies enrichment. First, we assigned each metQTL to its nearest gene. Secondly, we assigned each metQTL to all genes for whom the metQTL is in an enhancer region for [enhancer-gene pairs were obtained from the ENCODE’s Enhancer-to-Gene (E2G) database (Gschwind *et al.* 2023)]. Thirdly, we implemented a method combining enhancer-gene pairs, distance to closest gene, and information regarding metabolic roles of individual genes. Our third method utilized a decision tree that first evaluates whether the closest gene has a known metabolic role. We defined a gene as having a metabolic role if it is annotated within the Gene Ontology term GO_0008152 (“metabolic process”; https://amigo.geneontology.org/amigo/term/GO:0008152). If it is, we converted the metQTL to this metGene. If the closest gene was not a known metabolic gene, we examined whether the metQTL is in an enhancer as indicated by E2G, if not, we assigned metGene by nearest gene. If the metQTL wasn’t nearest to a known metabolic gene, and was in an enhancer region as indicated by E2G for a known metabolic gene, we assigned metGene to the gene with the highest E2G score (Fig. S4a). Indeed, the third method, combining multiple data sources, was the best at converting metQTLs to metGenes (Fig. S4b and c). After identifying metGenes, we determined metabolite–cell type associations by calculating average outlier expression, applying Benjamini-Hochberg adjustment to reduce false positives.

### 2.5 Fetus associations

scRNAseq was downloaded from Cao *et al.* (2020). Then, we applied our approach using 27 simple metabolites derived from UK Biobank metQTLs. To enable the comparison between adult and fetal samples, we manually identified pairs of cell types annotated the same across Tabula sapiens and Cao *et al.* (2020) by matching cell type names and pairing biologically equivalent annotations. For example, the fetal “β-cell (Pancreas)” annotation was matched to the adult cell type “type B pancreatic cell.” The adult and fetal datasets are comparable, as the adult Tabula Sapiens dataset used 500 000 cells from 24 tissues from 15 human donors, while the fetal Cao *et al.* (2020) data has 28 fetal donors samples representing 4 million cells.

## Supplementary Material

btag330_Supplementary_Data

## Data Availability

UKBiobank metQTL summary statistics are available via download from the GWAS Catalog accessions GCST90449363–GCST90451603, Table S12 (Tambets *et al.* 2026). TOPMed metQTL summary statistics are available used in this study are available through the database of Genotypes and Phenotypes (dbGaP) under accession number phs001974.v5.p1. Access to the data can be requested via the dbGaP website (https://www.ncbi.nlm.nih.gov/gap).

## Code availability

Code used in this work is available via Github at https://github.com/haimkru/Metabolite-Cell-Type-Associations.
