# Supplementary material for: Genomics-informed approach identifies which cell types regulate the metabolome
Source: Bioinformatics. 2026 Jun 2;42(6):btag330. doi: 10.1093/bioinformatics/btag330 (PMC13284994; doi:10.1093/bioinformatics/btag330)
Supplement: btag330_Supplementary_Data [file btag330_supplementary_data.zip › 3.pdf]

Extended Data Figures

UK Biobank compared to TOPMed metabolite-cell type associations

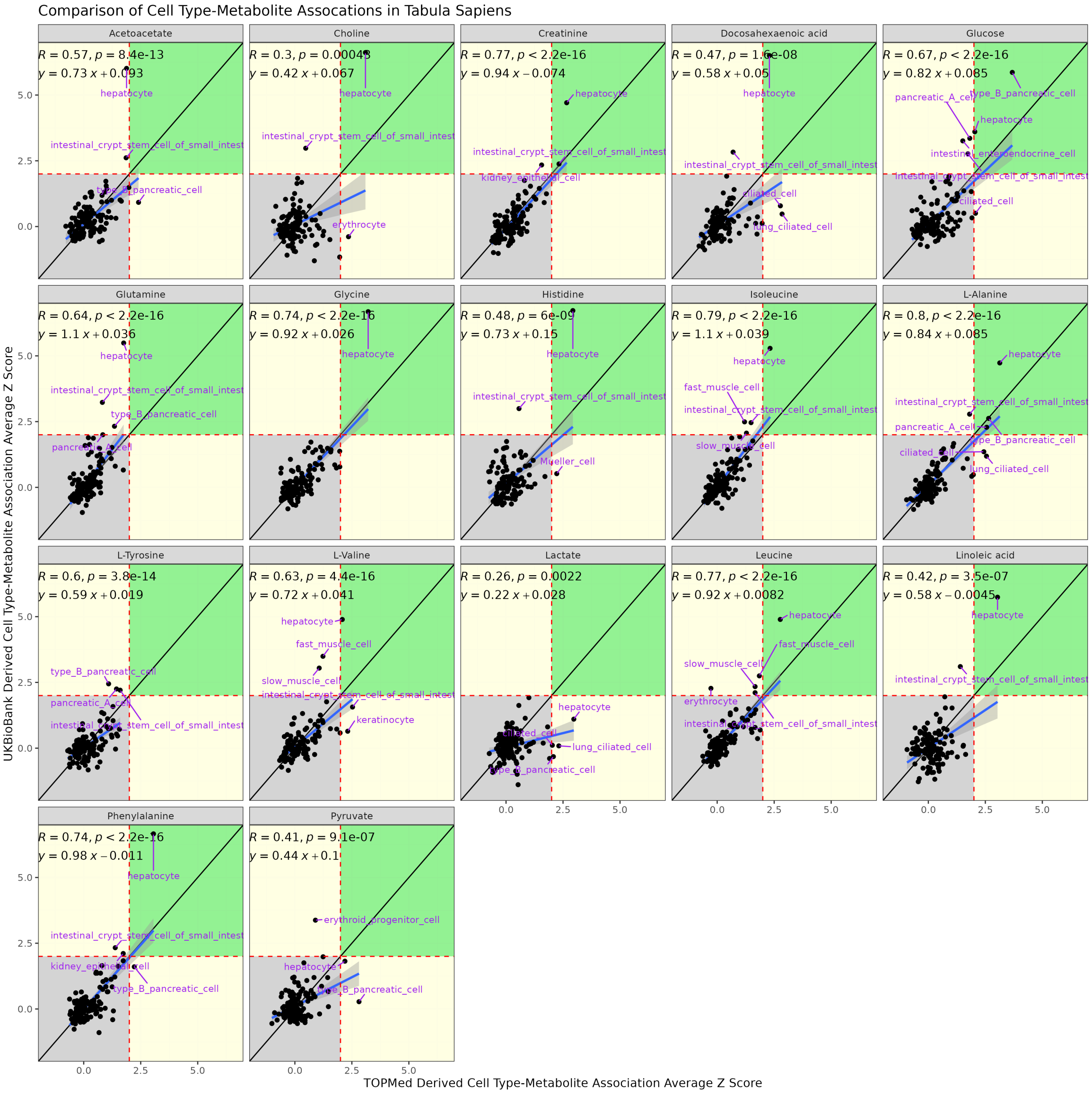

Extended Data Fig. 1 Comparison of association results for 17 metabolites found both data sources, derived from analyses conducted using TOPMed and UK Biobank metQTLs.
***This analysis was performed with Tabula Sapiens Adult scRNAseq.**

Robustness of metabolite-cell type associations across variability factors

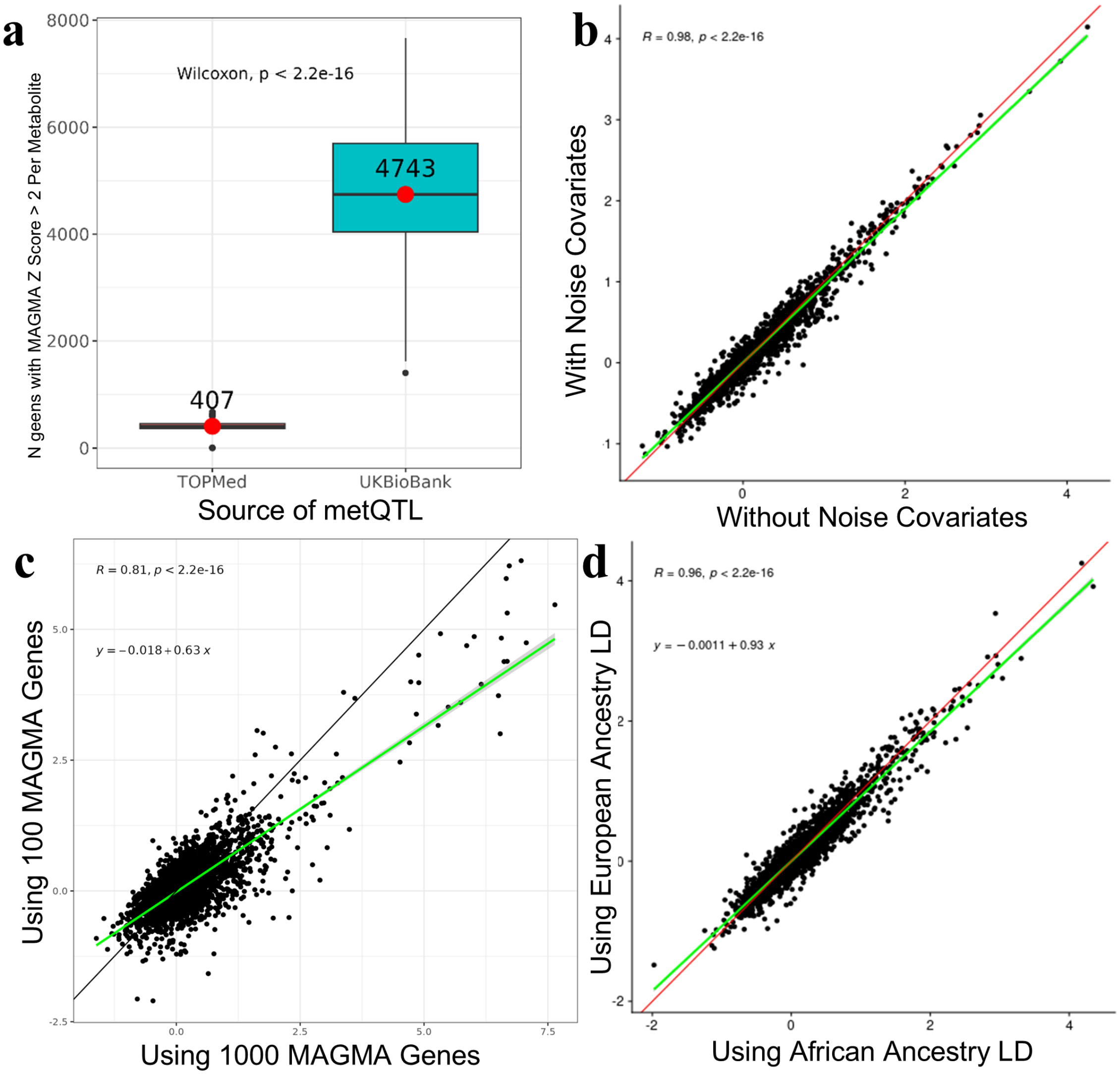

Extended Data Fig. 2 **a**, For each individual metabolite that is found in both UK Biobank and TOPMed, we performed gene-level association testing using MAGMA on metQTL data from each dataset. To compare the datasets, we quantified the number of significantly associated genes (defined by a MAGMA Z-score > 2) identified per metabolite. Overall, individual metabolites in TOPMed yielded fewer associated genes than those in the UK Biobank. This difference is likely driven by the UK Biobank's much larger sample size, which gives greater power to detect these gene-level associations. **b-d**. The effects of different variables on the association scores (average cell type's scDRS Z-scores) between metabolites and cell types were compared using 27 simple metabolites from the UK Biobank metQTL data together with the Tabula Sapiens adult scRNA-seq data. **b**, Comparison of association scores with and without the single cell quality variables of the number of features (nFeature) in cell, and number of reads (nCount). **c**, Comparison of association scores using the top 1000 metGenes versus using only the top 100 metGenes as derived from MAGMA. **d**, Comparison of association scores using Using MAGMA-derived metGene Z scores calculated with LD reference panels for African versus European ancestry, showing the LD reference does not largely affect metabolite-cell type associations. ***The plots include an ab line ($y = x + 0$).

Removing hepatocyte signals doesn't affect metabolite-non hepatocyte signals

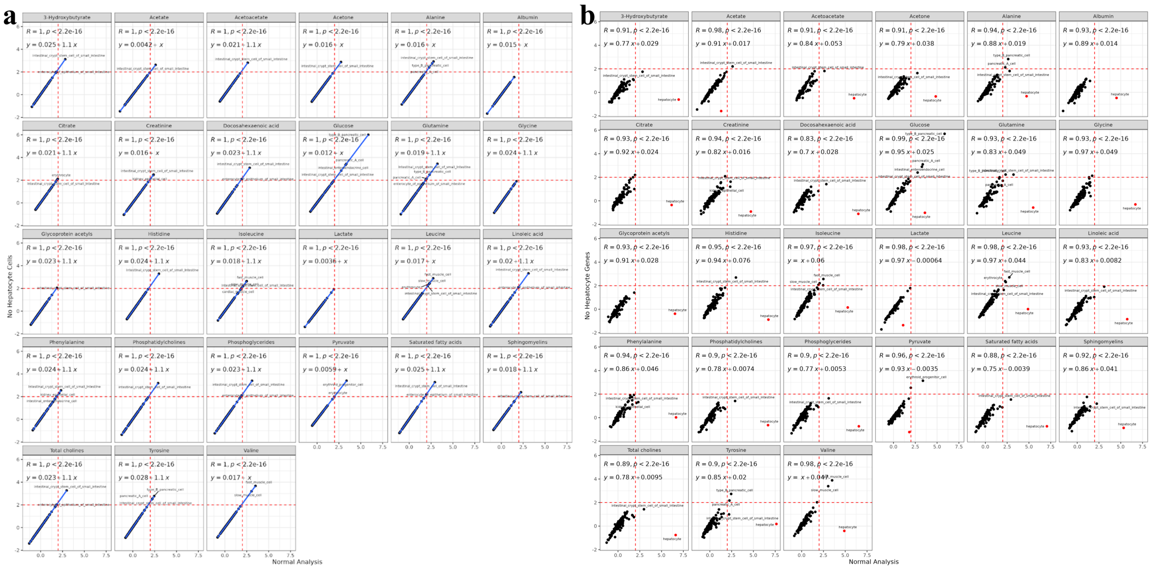

Extended Data Fig. 3 Comparisons of cell type – metabolite association results method normally versus alternative method for removing the hepatocyte signal (Methods **??**). Each dot represents a single metabolite-cell type association's average Z score. **a**, Results from the analysis in which hepatocyte cells are removed. **b**, Results from the analysis in which hepatocyte gene markers are removed. Hepatocytes weren't included in correlation.
*****This analysis was done using UK Biobank metQTL data integrated with Tabula Sapiens Adult scRNAseq.

metQTL to metGene Conversion via gene annotations and enhancers

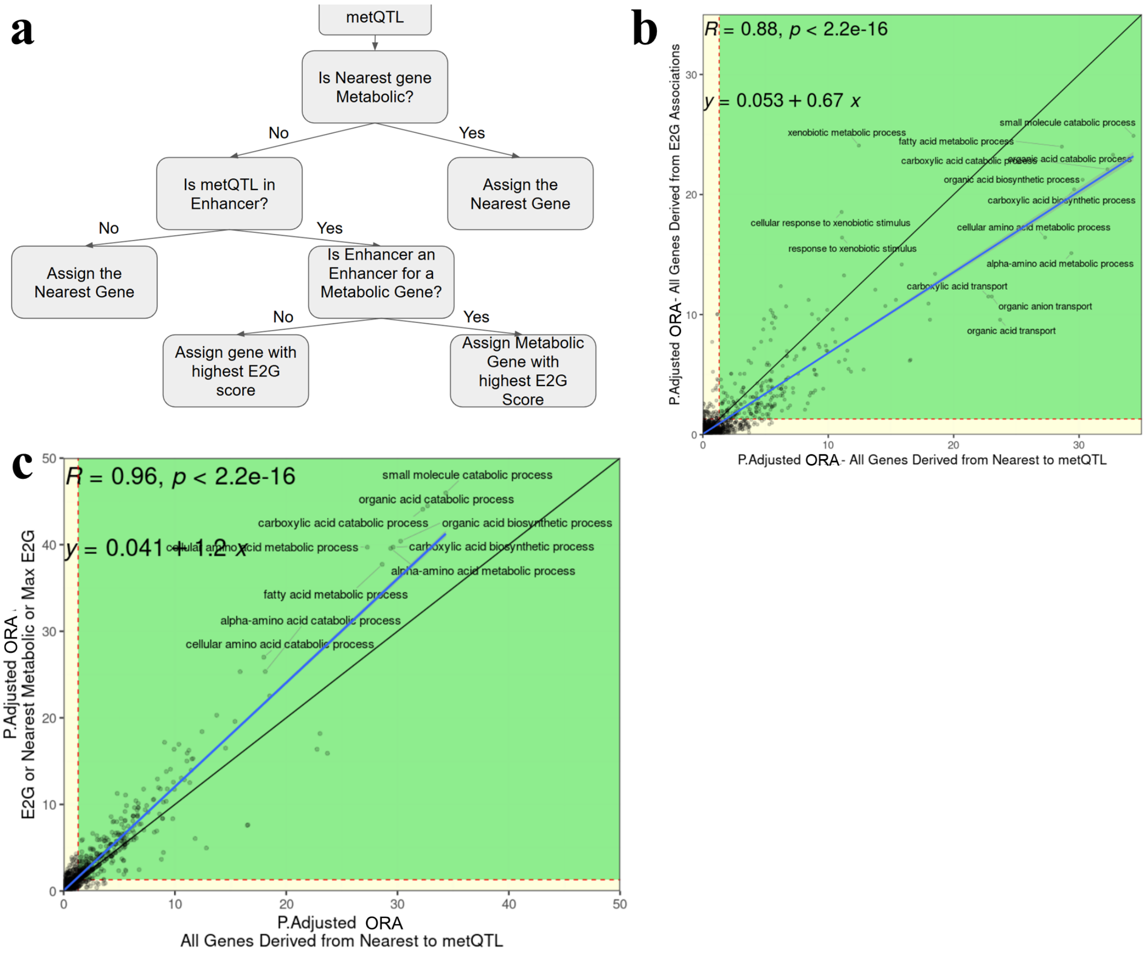

Extended Data Fig. 4 **a**, This panel outlines the pipeline for the combined method of linking metQTLs to genes, utilizing both gene annotations and E2G data (Methods **??**). **b**, The scatter plot displays the p-values from over-representation analysis (ORA) for Gene Ontology Biological Processes with the x-axis representing variant-to-gene mapping by the nearest gene, while the y-axis represents variant to gene mapping by assigning a metQTL to all genes associated with the enhancer in any cell type. **c**, The scatter plot displays the p-values from over-representation analysis (ORA) for Gene Ontology Biological Processes. The x-axis reflects ORA results via variant-to-gene mapping by the nearest gene, while the y-axis reflects ORA results via the combined method.
*This analysis was done using TOPMed metQTL data integrated with Tabula Sapiens Adult scRNAseq.
**Over-representation analysis (ORA) was performed using the clusterProfiler enrichGO function in R to identify enriched Biological Process terms from the org.Hs.eg.db and Gene Ontology databases. Statistical significance was calculated using a hypergeometric test, with p-values adjusted for multiple testing via the Benjamini-Hochberg (BH) method.[1]

Single gene metabolite-cell type associations

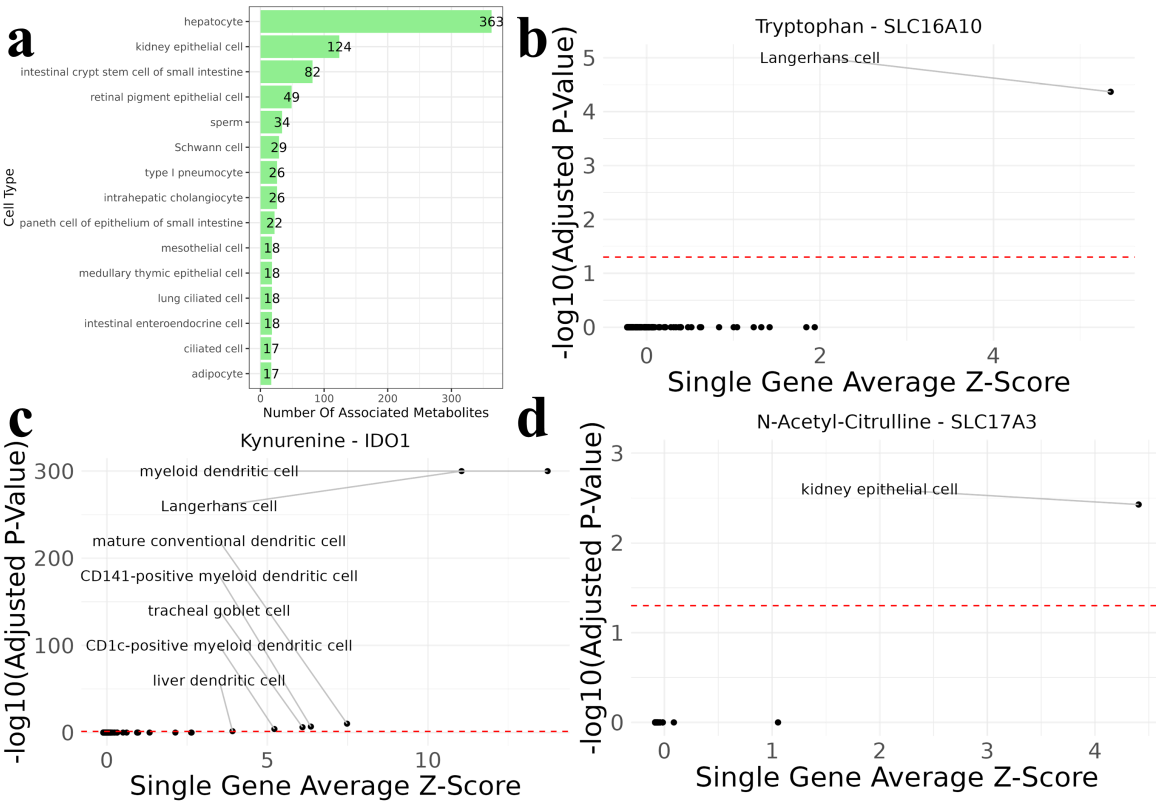

Extended Data Fig. 5 **a**, Number of metabolites for whom cell type is an expression outlier for associated metGene. **b**, Cell types expressing high levels of tryptophan associated gene SLC16A10. **c**, Cell types expressing outlier levels of Kynurenine associated gene IDO1. **d** Cell types expressing outlier levels of N-Acetyl-Citrulline associated gene SLC17A3.
*****This analysis was done using TOPMed metQTL data integrated with Tabula Sapiens Adult scRNAseq.

Fetus metabolite-cell type associations are similar to adult's

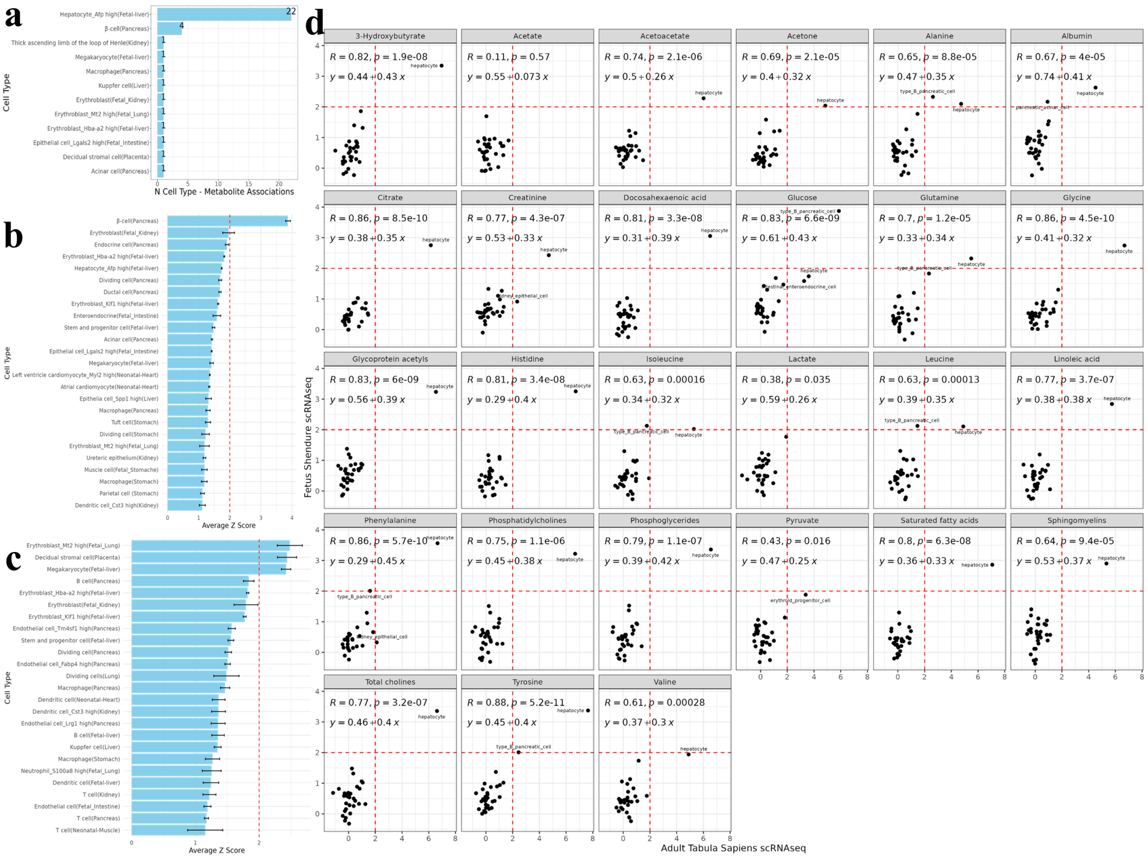

Extended Data Fig. 6 **a**, Total Fetal UK Biobank metabolite-cell type associations with lower Confidence interval of cell type average Z score > 1.96. Cell type associations with; **b**, Glucose. **c**, Lactic acid. d) Comparison of cell type-metabolite association results in all 27 simple metabolites (Methods **??**).
